# Berberine as a Potential Agent for the Treatment of Colorectal Cancer

**DOI:** 10.3389/fmed.2022.886996

**Published:** 2022-04-28

**Authors:** Xi Jiang, Zhongxiu Jiang, Min Jiang, Yan Sun

**Affiliations:** ^1^Department of Cardiology, Shengjing Hospital of China Medical University, Shenyang, China; ^2^Department of Oncology, Shengjing Hospital of China Medical University, Shenyang, China; ^3^Department of Gastroenterology, First Affiliated Hospital of China Medical University, Shenyang, China; ^4^Department of Gastroenterology, Shengjing Hospital of China Medical University, Shenyang, China

**Keywords:** berberine, colorectal cancer, antitumor, gut microbiota, mucosal barrier

## Abstract

Colorectal cancer (CRC) is one of the most commonly diagnosed and deadly malignancies worldwide. The incidence of CRC has been increasing, especially in young people. Although great advances have been made in managing CRC, the prognosis is unfavorable. Numerous studies have shown that berberine (BBR) is a safe and effective agent presenting significant antitumor effects. Nevertheless, the detailed underlying mechanism in treating CRC remains indistinct. In this review, we herein offer beneficial evidence for the utilization of BBR in the management and treatment of CRC, and describe the underlying mechanism(s). The review emphasizes several therapeutic effects of BBR and confirms that BBR could suppress CRC by modulating gene expression, the cell cycle, the inflammatory response, oxidative stress, and several signaling pathways. In addition, BBR also displays antitumor effects in CRC by regulating the gut microbiota and mucosal barrier function. This review emphasizes BBR as a potentially effective and safe drug for CRC therapy.

## Introduction

Colorectal cancer (CRC), one of the most common cancers worldwide, has been reported as the third most common cancer in men (10%) and second most common in women (9.2%) (1), accounting for the fourth most common cause of cancer-related death (2). It is a heterogeneous disease with multifarious, hereditary, and biological characteristics. Subtypes of CRC have been demonstrated to present different prognoses and therapeutic responses (3). The incidence rate of CRC has been decreasing in many developed countries; nevertheless, the prevalence has increased in adults younger than 50 years of age in numerous economically underdeveloped countries (4, 5). Although tremendous improvements have been made in the diagnosis and management of CRC, the prognosis remains poor (6). Currently, treatment for CRC mainly includes surgery, radiotherapy, and chemotherapy (7). For unresectable tumors, chemotherapy is a fundamental and indispensable therapeutic regimen. The common cytotoxic chemotherapy drugs include 5-fluorouracil (5-FU), irinotecan, oxaliplatin, raltitrexed, and lonsurf, as well as molecular-targeted agents, such as anti-vascular endothelial growth factor (VEGF) therapies (e.g., bevacizumab, Ziv-aflibercept, ramucirumab, and regorafenib) and anti-epidermal growth factor receptor (EGFR) drugs (e.g., cetuximab and panitumumab) (8). However, these cytotoxic chemotherapy drugs kill tumor cells by direct cytotoxicity and have poor selectivity for tumors; therefore, these drugs not only kill tumor cells but also kill normal cells (9). Moreover, the mucosal barrier tends to be disrupted by common chemotherapeutic drugs, initiating intestinal mucositis (IM). This disruption is unfavorable for the later treatment of CRC (10). In addition, chemotherapy usually causes gastrointestinal (GI) epithelium injury, which is mediated at least in part by the activation of an inflammatory cascade (10). Furthermore, several limitations and adverse effects have also been reported during molecular-targeted therapy. Molecular-targeted therapy is relatively expensive, and patients might require multiple targeted agents. Level 3 or level 4 adverse events can be infrequently detected, and their incidence might increase when two or more targeted agents are combined (11). Furthermore, CRC involves multiple pathways, whereas conventional drugs only target one aspect. In addition, there is a substantially increased economic burden due to management. Therefore, more effective treatment approaches and medical interferences with safety and high efficacy are urgently needed. Traditional Chinese medicine (TCM) has been extensively acknowledged as a typical complementary and alternative treatment in China for patients with tumors (12). As a typical Chinese herbal medicine-derived phytochemical, berberine (BBR) and its derivatives have attracted increasing attention in cancer treatment. The current review highlights several beneficial properties of BBR and could provide novel insights into CRC treatment (13, 14).

## Colorectal Cancer

### Epidemiology of Colorectal Cancer

According to a report from 40 European countries, an estimated 3.91 million new cases of cancer occurred in Europe in 2018, including 500,000 CRC cases. There were ~272,000 (13.2%) cases in men and 228,000 (12.3%) cases in women (15). In addition, 1.9 million incident cases and 0.9 million deaths worldwide were estimated to occur in 2020 (3). The overall incidence of CRC has been declining in many economically advanced countries; however, reports from the United States and other economically advanced countries, such as Australia, Canada, and Norway, have demonstrated that the prevalence is increasing in adults younger than 50 years of age (4, 5, 16–20). For example, the incidence of colon cancer increased by 1.8, 2.9, 2.9, and 3.1% per year in the United Kingdom (UK), New Zealand, Australia, and Denmark, respectively, in people younger than 50 years old, and the incidence of rectal cancer in the same age group also increased by 1.4, 2.6, and 3.4% per year in the UK, Australia, and Canada, respectively (20). Moreover, it has been reported that the incidence of colon cancer in individuals aged 20–29 years old increased by 9.3% every year from 2004 to 2014 in Australia, while the incidence of rectal cancer increased by 7.1% each year between 1993 and 2014 (17). The mortality of CRC has been reported to be decreased in many longstanding and economically advanced countries, such as the United States, Australia, New Zealand, most Western European countries (Austria and the UK.), some Asian countries (Japan), and Eastern European countries (the Czech Republic and Slovakia.) (21, 22). Effective prevention, early diagnosis through screening, interventions to reduce risk factors, and/or improvement of treatment strategies could contribute to reduced mortality (23). Nevertheless, mortality is still on the rise in both men and women in many low-income countries and regions, such as Mexico, Chile, and elsewhere in Latin America (21, 24, 25). CRC represents a huge burden globally on family and society and has a positive relationship with socioeconomic status (26–28). This burden is estimated to continue to increase due to the growth and aging of the population, as well as due to the adoption of high-risk behaviors and lifestyles, particularly in less economically developed countries (29, 30).

### Prevalent Sites and Risk Factors of Colorectal Cancer

**Figure 1:**
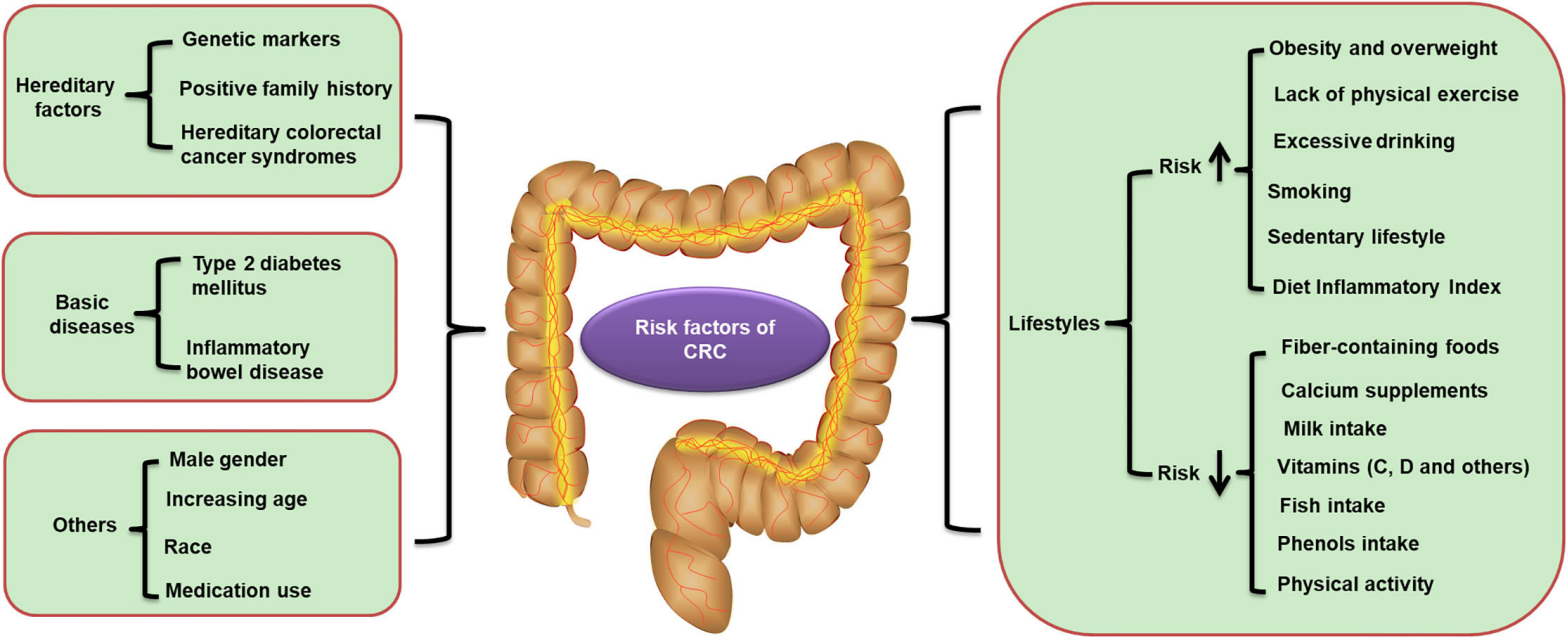
Risk factors of CRC. CRC, colorectal cancer.

Colorectal cancer is usually observed in the proximal colon (41%), distal colon (22%), and rectum (28%) (31). Nevertheless, there can be differences in the location depending on age and gender (32). It has been reported that the most frequent sites of CRC in young patients are the rectum and the sigmoid colon (33). Genetic and environmental risk factors have been demonstrated to play critical roles in the progression and development of CRC. A number of molecular genetic markers have been identified for the diagnosis, prognosis, and treatment of CRC, such as *KRAS, ERBB2, MLH*, and *NAV2/TCF7L* (34, 35). An increasing amount of evidence has supported associations between a positive family history of CRC and an increased risk for CRC (36–38). The reported heritability of CRC ranges from 12 to 35% according to twin and family studies (39, 40). Although tremendous advances have been made in the genome-wide association studies of CRC (41, 42), there remain many elusive factors affecting heritability (43). Hereditary colorectal cancer syndromes affect people with a high lifetime risk of developing CRC caused by an inherited or *de novo* germline mutation, accounting for 5–10% of all CRCs (44, 45). This subgroup can be divided into nonpolyposis and polyposis syndromes. The former includes Lynch syndrome, and the latter contains familial adenomatous polyposis (FAP), Peutz-Jeghers syndrome, and MUTYH-associated polyposis (MAP). Polyposis syndromes are more effortlessly diagnosed by doctors due to the number of polyps; however, nonpolyposis syndromes are commonly overlooked because these patients present few adenomas, and these adenomas are morphologically similar to sporadic lesions. Hence, systematic molecular investigation in subjects of any age or subgroup of subjects <70 years of age might improve the diagnosis of this genetic syndrome. Moreover, some basic diseases, such as inflammatory bowel disease (IBD) and type 2 diabetes mellitus (T2DM), are also well known to be associated with CRC (46, 47). In addition, several acquired environmental lifestyles have been recognized as important risk factors for CRC, such as obesity and overweight (48), lack of physical exercise (49), excessive drinking (50), smoking (51, 52), high Diet Inflammatory Index (DII) scores (53, 54), and sedentary lifestyles (55). Additionally, some factors, such as healthy diet structure (e.g., fiber-containing foods, calcium supplements, milk intake, vitamins, fish intake, and phenol intake) and physical activity, have been described to reduce the risk factors of CRC (56–59). However, some of the data have been inconsistent, and the effect on rectal cancer vs. colorectal cancer may also be different. Furthermore, healthy lifestyle habits have been shown to improve the prognosis and mortality of CRC survivors (60, 61). In addition, male sex, increasing age, race, and medical intake have also been demonstrated to play critical roles in the progression and development of CRC (62–64) (Figure 1).

### Carcinogenic Pathways of Colorectal Cancer

**Figure 2:**
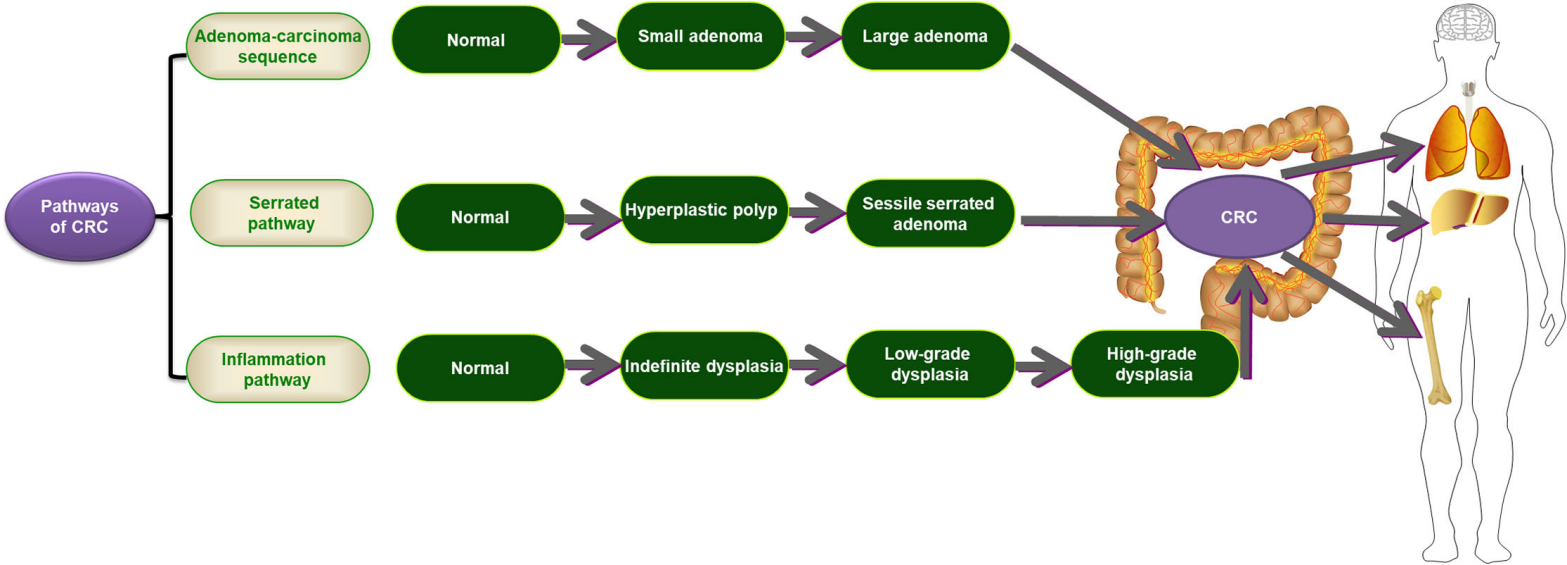
The three distinctive oncogenic pathways involving CRC. CRC, colorectal cancer.

There are three distinctive oncogenic pathways involved in CRC, including the adenoma-carcinoma sequence, serrated pathway, and inflammatory pathway (Figure 2). The adenoma-carcinoma sequence is a typical pathway that can explain ~60–90% of sporadic CRC. In this classic pathway, normal cells are driven to transfer to small adenomas, then to large adenomas, and finally to malignancies by the gradual accumulation of genetic and epigenetic changes (65). Several factors have been confirmed to play imperative roles in this pathway, including male sex, increasing age, positioning in the distal colon, tobacco and alcohol use, and a high-fat diet (66, 67). This pathway is primarily characterized by chromosomal instability (CIN) and is related to the development of the CIN-positive subtype. Inactivating mutations in adenomatous polyposis coli (*APC*), a well-known tumor suppressor gene, have been demonstrated in more than 70% of adenomas and CRCs (68, 69). In addition, some mutations in other genes, such as *KRAS, TP53, SMAD4*, and *PIK3CA*, also contribute to this model (70, 71). Recently, the serrated pathway was described as one of the CRC subsets, accounting for ~10–15% of sporadic CRCs (30). In this model, serrated polyps are considered precursor lesions to CRC, and they are characterized by the transformation of normal cells to hyperplastic polyps (HPs), then to sessile serrated adenoma (SSA), and ultimately to CRC (72, 73). The *BRAF* mutation and CpG island methylator phenotype (CIMP) have significant involvement in this pathway (72, 74). The inflammatory pathway is another distinctive oncogenic pathway involved in CRC. It is highlighted by the progression of normal cells to indefinite dysplasia, then to low-grade dysplasia, next to high-grade dysplasia, and finally to CRC, stimulated by chronic inflammation (75). However, this pathway only accounts for <2% of all CRCs (30, 76). IBD, especially ulcerative colitis (UC), is representative of this pathway. Unlike the above two precursor lesions, chronic inflammation-induced dysplasia usually appears as a flat mucosa with multifocality. Different from the adenoma-carcinoma sequence, mutations of *TP53* are an early event in this model (75).

### Management of Colorectal Cancer

Endoscopic resection can be performed as a minimally invasive method for early-stage colon cancer. It includes endoscopic mucosal resection (EMR), endoscopic submucosal dissection (ESD), and endoscopic full-thickness resection. The choice of endoscopic resection method depends on the size of T1 cancers. However, some individuals with T1 cancers who experience endoscopic resection require additional colectomy with lymph node dissection due to lymph node metastases (77). Surgery is the cornerstone and one of the most effective management options for CRC. However, the surgical approach for colon cancer is dependent on the tumor location and anatomic relationship with blood vessels. Currently, laparoscopy has become a standard method for CRC in many countries. Compared to conventional open surgery, laparoscopic resection is a minimally invasive technique that can expedite gastrointestinal function recovery and reduce the length of hospital stay, particularly for elderly patients (78). In addition, it has been shown that laparoscopic resection is associated with reduced postoperative mortality, and there are no adverse effects on long-term survival (79, 80). More recently, robotic colectomy has emerged as a popular method for the treatment of CRC (81). Although robotic colectomy can decrease the length of stay, it costs more and requires a longer operation time than laparoscopic colectomy, and no significant differences have been observed in perioperative and short- or long-term outcomes (82, 83). In addition to surgery for CRC, several drugs have been permitted for CRC management in clinical trials. The Food and Drug Administration (FDA)-approved anticancer drugs include 5 cytotoxics (5-FU, capecitabine, TAS-102, irinotecan, and oxaliplatin) and eight biologics/targets (cetuximab, panitumumab, bevacizumab, Ziv-aflibercept, regorafenib, ramucirumab, pembrolizumab, and nivolumab). As a standard first-line chemotherapy drug, 5-FU plays significant role in palliative and adjuvant systemic therapy for CRC. Nevertheless, some adverse effects of these drug treatments have been reported, such as diarrhea, gastrointestinal tract injury, stomachache, and fever (84–86). Compared to chemotherapy, immunotherapy could prompt the memory function of the adaptive immune system and trigger the immune system against tumors, achieving long-term durable responses. Furthermore, fewer adverse effects have been observed in immunotherapy because of immune tolerance.

### Berberine

#### Source and Metabolites of Berberine

Berberine, also known as *Coptis rhizome*, is a pentacyclic isoquinoline alkaloid. It can be found in the Chinese herb *Coptis chinensis* and numerous Berberis plants, such as *Berberis aristata* (81), *Berberis vulgaris* (87), and *Berberis darwinii* (88). The molecular formula of BBR is C_20_H_18_NO_4_, and the molecular weight is 336.337 g/mol. The isoquinoline alkaloids in Huanglian extracts include BBR, xanthophylline, epiberberine, and pharmacophorine. BBR is mainly metabolized in the intestine and liver (89, 90). With the involvement of cytochrome and UDP glucuronosyltransferase (UGT), BBR could be metabolized in liver cells (91). There are four major types of BBR metabolites: berberrubine, thalifendine, demethyleneberberine, and jatrorrhizine (92). However, the number of metabolites varies by species. For example, 16 and 11 metabolites were identified in rats (93) and mice (91), respectively.

#### Pharmacological Effects of Berberine

Berberine has been used as an important TCM for a long period of time owing to its extraordinary efficiency, such as in alleviating fever, dispelling fire, drying dampness, cooling blood, and detoxifying toxins. Recently, an increasing number of studies have reported regarding the novel pharmacological effects of BBR. For instance, BBR exerts remarkable anti-inflammatory (94–96), antiviral (97), antioxidant (98), antidiabetic (99), immunosuppressive (100), cardiovascular (101, 102), and neuroprotective (103) activities. Therefore, BBR could be utilized to treat various disorders (104–107), including metabolic, cardiovascular, digestive, and neurological diseases. It has been demonstrated that BBR displays protective effects against digestive diseases by inhibiting toxins and bacteria and fortifying the intestinal mucosa (108). Moreover, BBR has been confirmed to regulate glucolipid metabolism, ameliorate energy consumption, and decrease body weight (106). Furthermore, BBR also presents strong cardiovascular protection and neuroprotective effects by improving cardiovascular hemodynamics, reducing hypertension, and attenuating atherosclerosis progression (101, 109).

#### Antitumor Effects of Berberine

Emerging evidence has shown that BBR exerts anticancer effects in several malignancies (110–112). BBR has been reported to inhibit cancer cell proliferation by affecting the cell cycle and autophagy and stimulating cell apoptosis. For example, BBR could induce G1 cycle arrest in A549 lung cancer cells by decreasing the levels of cyclin D1 and cyclin E1 (113). BBR could inhibit the expression of cyclin D1 in HepG2 liver cancer cells (114). BBR also induced G1 cycle arrest by inhibiting cyclin B1 expression and CDC2 kinase in some cancer cells (115). Moreover, BBR has been suggested to induce autophagy in glioblastoma by targeting the AMP-activated protein kinase (AMPK)/mechanistic target of rapamycin (mTOR)/ULK1 pathway (116) and in liver cancer cells by stimulating the release of beclin-1 from the Bcl-2/beclin-1 complex (117). In addition, BBR has been revealed to stimulate apoptosis in leukemia by upregulation of caspase-8 and caspase-9 (118) and in skin squamous cell carcinoma A431 cells by increasing cytochrome C levels (119). In addition, BBR has been confirmed to inhibit cell migration and invasion by inhibiting the expression of epithelial–mesenchymal transition (EMT) and metastasis-related proteins, such as matrix metalloproteinases (MMPs) and E-cadherin, the tumor microenvironment, and/or the caspase-1/interleukin (IL)-1β and nuclear factor kappa B (NF-κB) signaling pathways (120–123). Interestingly, canadine, a derivative of flavopiridol, has been shown to improve cancer-induced muscle wasting (124). Furthermore, BBR has shown antitumor effects by interacting with microRNAs (125) and inhibiting telomerase activity (126).

#### Molecular Mechanism of Berberine in the Treatment of Colorectal Cancer

**Figure 3:**
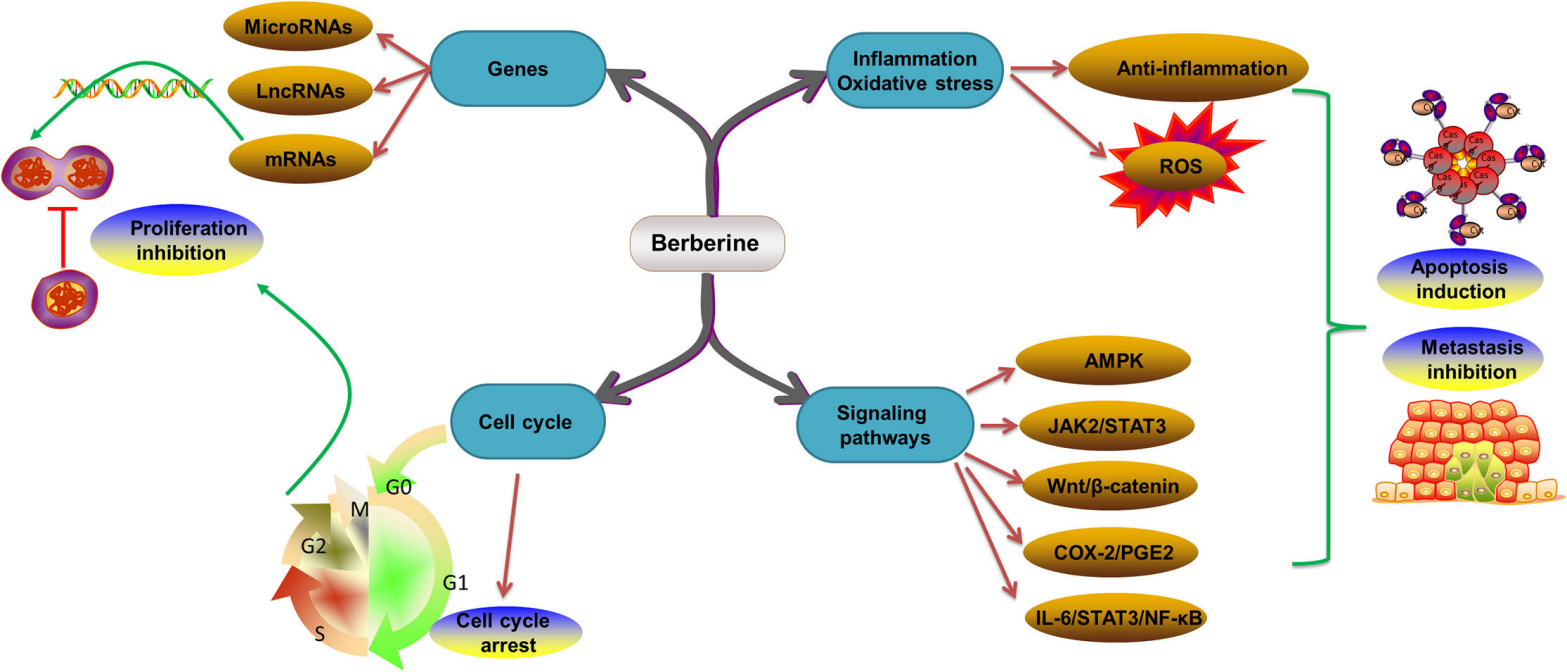
Molecular mechanism of BBR in the treatment of CRC. CRC, colorectal cancer; BBR, berberine; ROS, reactive oxygen species; AMPK, AMP-activated protein kinase; STAT3, signal transducer and activator of transcription 3; COX-2, cyclooxygenase-2; PGE2, prostaglandin E2; IL-6, interleukin 6; NF-κB, nuclear factor kappa B.

Numerous studies have revealed that BBR is a safe and effective treatment for CRC (127–129). The reported mechanisms include regulation of gene expression (microRNAs, long noncoding RNAs (lncRNAs), and mRNAs) (130–133), growth factors (EGFR) (134), cell cycles (135), signaling pathways (AMPK, JAK2/signal transducer and activator of transcription 3 (STAT3), Wnt/β-catenin, IL-6/STAT3/NF-κB, and cyclooxygenase-2 (COX-2)/prostaglandin E2 (PGE2) pathways) (13, 129, 136), inflammation, and oxidative stress (Figure 3). The effects and molecular mechanism of BBR in the treatment of CRC are summarized in Table 1.

**Table 1:** The effects and molecular mechanism of BBR in the treatment of CRC.

**Authors and references**	**Publish year**	**Cell line/animals/tissues**	**Effect of BBR**	**Mechanism**	**Experimental model**
Zhang (130)	2020	HCT116 and SW480 cell lines, male BALB/c nude mice	Anti-proliferation, induces apoptosis and cell cycle arrest	Downregulates IGF2BP3	*In vitro* and *in vivo*
Gong (131)	2020	SW480 and HT-29 cell lines	Anti-proliferation, anti-migration, and induces apoptosis	Downregulates GRP78	*In vitro*
Liu (132)	2016	*In vitro* culture of colorectal tissue	/	Reduces miR-429, E-cadherin, and Par3	*In vitro*
Dai (133)	2019	HT29 and HCT116 cell lines	Promotes apoptosis	Increases lncRNA CASC2	*In vitro*
Wang (134)	2013	IMCE and HT-29 cell lines, nude mice, and *APC* ^min/+^ mice	Anti-proliferation, induces apoptosis and cell cycle arrest	Downregulates EGFR and activates Cbl	*In vitro* and *in vivo*
Samad (135)	2021	HCT116 cell line	Anti-proliferation, inhibits telomerase activity and induces cell cycle arrest and telomere Erosion	Increases CCND1 and downregulates CDK4, TERT, and TERC	*In vitro*
Liu (136)	2015	SW620 and LoVo cell lines, male BALB/C nude mice	Anti-proliferation, inhibits invasion and metastasis	Reduces levels of COX-2/PGE2, phosphorylation of JAK2 and STAT3, and expression of MMP-2/-9.	*In vitro* and *in vivo*
Lü (137)	2018	HCT116 cell line	Anti-proliferation, and induces apoptosis	Regulates the microRNA-21-ITG?4-PDCD4 axis	*In vitro*
Su (129)	2015	HCT-15, HCT116WT, and HT-29 cell lines	Anti-proliferation	Enhances the antitumor activity of NVP-AUY922	*In vitro*
Dai (138)	2019	HT-29, HCT116, SW480, SW620 and LoVo cell lines	Anti-proliferation, promotes apoptosis, and inhibits migration and invasion	Increases lncRNA CASC2 and regulates EZH2/Bcl-2 axis	*In vitro*
Huang (139)	2017	Primary culture of colon tissues from neonatal rats	Anti-proliferation	Mediates the expression of DNMTs and target miRNAs	*In vitro*
Soffar (140)	2019	HCT116 spheroids	Anti-proliferation, induces cell cycle arrest	Induces G1-phase cell cycle delay and decreases the S-phase fraction of cells	*In vitro*
Liu (141)	2020	DLD-1 and Caco-2 cell lines	Anti-proliferation, induces cell cycle arrest	Inhibits the SCAP/SREBP-1 signaling pathway-mediated lipogenesis	*In vitro*
Li (142)	2015	HCT116, SW480 and LOVO cell lines, female FVB mice	Anti-inflammation, anti-proliferation, and induces apoptosis	Suppresses COX-2 expression via regulating AMPK pathway	*In vitro* and *in vivo*
Li (143)	2017	IMCE, RAW 264.7 macrophages, and HCT116 cell lines, C57BL/6J-*APC*^min/+^ mice	Anti-inflammation, and anti-proliferation	Interferes with inflammatory response-driven EGFR signaling pathway	*In vitro* and *in vivo*
Wu (144)	2012	HCT116 cell line, female SD rats	Anti-proliferation, induces apoptosis and cell cycle arrest	Targets Wnt/β-catenin signaling pathway	*In vitro* and *in vivo*
Deng (145)	2022	C57BL/6 male mice	Anti-inflammation, improves intestinal barrier function, modulates gut microbiota dysbiosis	Inhibition of the JNK/STAT3 and β-Catenin pathways	*In vivo*

*BBR, berberine; CRC, colorectal cancer; IGF2BP3, insulin like growth factor 2 mRNA binding protein 3; GRP78, glucose-regulated protein 78; miR, microRNA; lncRNA, long non-coding RNA; CASC2, cancer susceptibility candidate 2; EGFR, epidermal growth factor receptor; Cb1, cannabinoid receptor 1; CCND1, cyclin D1; CDK4, cyclin-dependent kinase; TERT, telomerase reverse transcriptase; TERC telomerase RNA component; COX-2, cyclooxygenase-2; PGE2, prostaglandin E2; STAT, signal transducer and activator of transcription; MMP, matrix metalloproteinase; ITG?4, integrin subunit beta 4; PDCD4, programmed cell death 4; EZH2, zeste 2 polycomb repressive complex 2 subunit; DNMTs, DNA methyltransferases; SCAP, SREBP cleavage-activating protein; SREBP1, sterol-regulatory element binding protein 1; AMPK, AMP activated protein kinase; SD, sprague dawely*.

#### Effects of Berberine on Gene Expression and Cell Cycle in Colorectal Cancer

Abnormal expression of miRNAs, lncRNAs, and mRNAs has been implicated in cancer development, including CRC. Based on the recently established evidence, BBR shows important regulatory effects on gene expression that are dysregulated in CRC, making it a potential agent for managing cancers. For example, BBR was reported to regulate the miR-21/integrin β4 (ITGβ4)/programmed cell death 4 (PDCD4) axis to exert anticancer effects on the CRC cell line HCT116 (137). In addition, BBR could decrease the expression levels of miR-429 while upregulating the expression of E-cadherin and partitioning defective 3 (Par-3) in CRC (132). Moreover, the combination of NVP-AUY922 and BBR induced cell growth arrest by suppressing cyclin-dependent kinase 4 (CDK4) expression and inducing miR-296-5p-mediated inhibition of the peptidylprolyl cis/trans isomerase NIMA-interacting 1 (Pin1)-β-catenin-cyclin D1 signaling pathway in CRC (129). In addition, BBR has been confirmed to prevent the proliferation and migration of CRC cells by decreasing glucose-regulated protein 78 (GRP78) (131). Furthermore, the lncRNA cancer susceptibility 2 (CASC2)/zeste 2 polycomb repressive complex 2 subunit (EZH2) /Bcl-2 axis was identified in BBR-induced CRC cell apoptosis (138). Huang et al. also suggested that DNA (cytosine-5)-methyltransferase PliMCI (DNMTs) and its targeted miRNAs were involved in the therapeutic effects of BBR on CRC (139). The cell cycle is an extremely conserved, ordered, and complex process that controls DNA replication and mitosis. It is regulated by many mechanisms to ensure that correct cell division occurs (146). Several reports have suggested that BBR can induce cell cycle arrest to inhibit cancer development. For instance, Huang et al. demonstrated that BBR induced G0/G1 cell cycle arrest in CRC and then further inhibited cell proliferation (130). Samad et al. found that BBR could also cause G0/G1 cell cycle arrest by regulating CCDN1 and CDK4 in the CRC cell line HCT116 and inhibiting telomerase activity (135). Soffar suggested that BBR diminished CRC cancer cell growth by modulating G1-phase cell cycle delay (140). In addition, Liu and coauthors observed that BBR suppressed cell proliferation by inducing G0/G1 phase cell cycle arrest in CRC cells (141). Therefore, the anti-proliferation effect of BBR might largely contribute to G0/G1 cell cycle arrest in CRC.

#### Effects of Berberine on Inflammation, Oxidative Stress, and Signaling Pathways in Colorectal Cancer

An important relationship has been confirmed between chronic inflammation and CRC development. It has been estimated that ~15–20% of cancer-related deaths involve an inflammatory response (147). CRC is colitis-associated cancer and patients with Crohn's disease (CD) or UC who develop CRC present an unfavorable prognosis (148, 149). This phenomenon might be explained by the chronic inflammatory response in the intestinal tract being able to trigger tumorigenesis and stimulate malignancy development (13). In addition, oxidative stress also plays a very important role in IBD carcinogenesis (66). During inflammation, activated inflammatory cells (neutrophils, macrophages, etc.) produce high levels of reactive oxygen species (ROS), including superoxide radicals, hydroxyl radicals, and hydrogen peroxide, which are important substances that contribute to tumorigenesis. Oxygen radicals can lead to abnormal DNA and RNA synthesis, and abnormal protein assembly and DNA repair (150). Moreover, oxygen radicals can also cause microsatellite instability (MSI) (151) and hypermethylation (152). In addition, oxygen radicals can activate genes that promote the production of free radicals, such as nitric oxide (NO) synthase and COX-2, allowing for a progressive inflammatory response and carcinogenesis (153). Therefore, inhibition of the inflammatory response and oxidative stress are beneficial for cancer treatment. BBR was reported to inhibit azoxymethane and dextran sulfate sodium (AOM/DSS)-induced CRC in a mouse model by suppressing COX-2 expression and regulating the AMPK pathway (142). Another discovery indicated that BBR inhibited the migration and invasion of CRC cells *via* the COX-2/PGE2-mediated JAK2/STAT3 signaling pathway (136). Additionally, Li and coauthors confirmed that BBR inhibited colitis-associated CRC by inhibiting inflammatory responses and subsequently suppressed EGFR signaling-involved tumor cell growth (143). Furthermore, BBR exerts its anti-inflammatory role in UC by modulating the IL-6/STAT3/NF-κB signaling pathway (154). BBR was described to inhibit the proliferation of CRC cells by inactivating the Wnt/β-catenin signaling pathway (144). Thus, BBR could be used as a critical anti-inflammatory and antioxidant agent in CRC.

### Gut Microbiota, Mucosal Barrier, and Colorectal Cancer

#### Gut Microbiota and Colorectal Cancer

The colon is covered with a mass of microorganisms. It has been reported that more than 500–1,000 species and a total of 10^13^ bacteria settle in the colon and other parts of the large intestine of adult humans. The diversity and composition of the microbiota could be changed by a variety of factors, such as age, dietary habits, pharmacotherapy, and psychological stress. (155, 156). The intestinal microflora is often considered an important organ acquired by the human body, which can protect the host from pathogenic bacteria, promote the host's digestion and absorption, affect drug metabolism and carcinogenesis, influence the absorption and distribution of fat, and regulate energy metabolism and the innate and acquired immune systems (157). Dysbiosis is an imbalance in the function or structure of gut microbiota and has been responsible for many disorders, including CRC. Noteworthy changes have been reported in specific bacterial clusters in CRC subjects (158). For instance, commensal bacterial species (such as *Faecalibacterium, Blautia*, and *Roseburia*) are found to be decreased, while harmful bacterial inhabitants (such as *Akkermansia, Fusobacterium nucleatum*, and *Clostridium difficile*) are extensively enriched in CRC (159). Dysbiosis in the gut microbiota could contribute to the development of CRC by modulating several different mechanisms, such as the inflammatory response, immune regulation, DNA damage, and the production of metabolites responsible for cancer development or suppression (160–164). Beneficial bacteria might compete for attachment sites to reduce the abundance of pathogenic bacteria and avoid infection. However, the pathogenic bacteria could increase intestinal permeation and are very closely related to the colon inflammatory response, which might be an important issue for the promotion of CRC (165). Furthermore, microbial metabolites, such as short-chain fatty acids (SCFAs), might also participate in the development of CRC. SCFAs have been reported to regulate the differentiation of Th1/Th17 cells and production of IL-10 by increasing the expression of transcription factor B lymphocyte-induced maturation protein 1 (Blimp-1) to maintain intestinal homeostasis (166). In addition, *Fusobacterium nucleatum* changed microbial structures and activated JAK-STAT and mitogen-activated protein kinase (MAPK) pathways, promoting the release of inflammatory factors (128). Moreover, the gut microbiota causes immune repair by modulating the intestinal barrier.

#### Effects of Berberine on Gut Microbiota and Mucosal Barrier

Common chemotherapeutic drugs tend to disrupt the mucosal barrier, which is detrimental to the late treatment of CRC (10). In addition, chemotherapy generally induces gastrointestinal (GI) epithelium damage that is at least partially intermediated by the activation of the inflammatory cascade (10). For example, 5-FU, a standard first-line treatment for CRC individuals, promotes apoptosis of epithelial cells and inhibits mucosal proliferation, while apoptotic cells can further promote inflammation. In contrast, BBR can inhibit tumor growth through meditation of the intestinal flora and mucosal barrier, and generally and ultimately improve weight loss (167). Moreover, BBR has been reported to modulate the composition of intestinal flora and significantly reduce flora diversity. For instance, BBR was demonstrated to inhibit the relative abundance of some of the intestinal flora, such as *Desulfovibrio, Lactobacillus acidophilus, Eubacterium, Lactococcus lactis*, and *Bacteroides* (168, 169), while it enriched *Bacteroides* in the colon and terminal ileum of C57BL/6 mice (169). In addition, it has been also demonstrated that BBR significantly reduced the relative abundances of both *Firmicutes* (such as *Lactobacillus* sp.) and *Bacteroidetes* in the gut of high-fat diet (HFD)-fed mice, indicating that the antimicrobial activity of BBR may be responsible for its anti-obesity effects (170). *Akkermansia muciniphila* (*A. muciniphila*) is a member of beneficial microbiota and a dedicated intestinal mucin degrader (171). Abnormal production and expression of mucin damage the mucinous layer, bringing bacteria into close contact with the intestinal epithelial cells and possibly triggering adverse host response and subsequent CRC development (172). BBR has been revealed to increase the growth of the populations of the symbiotic genus *Akkermansia* (173) and may further have effects on mucin expression. Moreover, BBR could modulate the gut microbiota in the colon by impacting the Treg/Th17 balance, reducing the levels of proinflammatory factors, including IL-17, IL-21, IL-22, IL-23, and IL-25, and increasing the levels of anti-inflammatory factors, such as IL-10 (174). In addition, BBR reduced bacterial endotoxins in the blood and alleviated the inflammatory response (175). Furthermore, it has been demonstrated that BBR decreases mucosal damage by modulating the levels of polyamines. Wu et al. (176) found that BBR could downregulate the levels of polyamine metabolism-associated proteins, including ornithine decarboxylase (ODC), c-myc, and hypoxia-inducible factor 1 subunit alpha (HIF-1α), and upregulate the levels of polyamine metabolic enzymes, including ornithine decarboxylase antizyme 1 (OAZ1) and spermidine/spermine N1-acetyltransferase (SSAT). At the same time, BBR could decrease the permeability of intestinal mucosa by modulating zonula occludens-1 (ZO1) and occludin (OCLN) (176). More recently, Deng and coauthors found that preadministration of BBR suppressed CRC development by inhibiting inflammation and proliferation and maintaining intestinal homeostasis (145).

#### Indoleamine-2, 3-Dioxygenase, Colorectal Cancer, and Berberine

Indoleamine 2,3-dioxygenase (IDO) is a key inflammatory cytokine-inducible rate-limiting enzyme of tryptophan catabolism, which includes three types, namely, IDO1, IDO2, and tryptophan 2,3-dioxygenase (TDO2). IDO has been demonstrated to play significant roles in the inhibition of intracellular pathogen replication and immunomodulation (177, 178). IDO is constitutively expressed in several human and mouse cells. It can catalyze the oxidative catabolism of tryptophan to kynurenine (179). In addition, IDO1 exerts its immunosuppressive effect by inhibiting the macrophage response and effector T cells by tryptophan starvation of sensitive T cells or accumulation of toxic metabolites (kynurenine) produced by tryptophan metabolism, subsequently inducing cell cycle arrest and effector T-cell death in the tumor microenvironment (180). In addition, the activity of IDO1 has been reported to directly stimulate cancer growth and proliferation by the production of kynurenine and the activation of β-catenin signaling (181). Previous studies discovered that IDO1 was highly expressed in CRC and was also correlated with impaired clinical outcomes (182–185). Therefore, targeting IDO1 for the treatment of cancer could be considered as an immunosuppressive-targeted strategy (177). Interestingly, BBR revealed uncompetitive and reversible inhibitory activity on IDO1, which might be due to direct binding to heme iron or occupation of the presumed tryptophan-binding site (186). Recently, a series of novel BBR derivatives targeting IDO1 has been designed to reduce the activity of IDO1. Wang et al. observed that compounds 2i and 2n of BBR displayed anticancer activity by increasing the specific lysis of natural killer (NK) cells to A549 cells through IDO1 (187). Both compounds repressed interferon (IFN)-γ-induced IDO1 expression *via* activation of AMPK and inhibition of STAT1 phosphorylation. Nevertheless, there have been no related studies about the effects of BBR on CRC *via* inhibition of IDO1 activity. Hence, compounds 2i and 2n could be selected as IDO1 modulators for small-molecule CRC immunotherapy for further investigation.

#### Limitations and Side Effects of Berberine

Although BBR presents valuable and promising biological effects in the management of cancers, the side effects of BBR cannot be overlooked (188). BBR should not be administered to infants with jaundice, pregnant women, or nursing women because of the potential risk of bilirubin-induced brain injury (189). In addition, intravenous administration of BBR can cause allergic reactions (190). Additionally, arrhythmia has been reported in hypervagotonic individuals after the administration of BBR (191). Other side effects, including nausea, cramping, diarrhea, flatulence, vomiting, rash, fever, constipation, and stomachache, have also been reported (192–194). Furthermore, a high dosage of BBR could cause low blood pressure, dyspnea, flu-like symptoms, and cardiac injury (190). Moreover, BBR can regulate the activity of P-glycoprotein (P-gp), and potential drug-drug interactions (DDIs) are observed when BBR is coadministered with P-gp substrates (195–197). For example, BBR in combination with P-gp inhibitor tetrandrine (Tet) can significantly improve the pharmacokinetics and hypoglycemic efficacy of BBR (196). Codelivery of the P-gp inhibitor tariquidar and BBR reversed the multiple drug resistance (MDR) in the K562/DOXO cell line (198). In addition, the efficiency of BBR is limited by its low bioavailability due to its poor absorption rate in the gut, low solubility in water, and fast metabolism. Studies have shown that the oral bioavailability of BBR is 0.68% in rats (199). Therefore, searching for novel methods to improve the absorption of BBR in the gut might be beneficial for the treatment of cancer. Previous studies have suggested complexation with C60 fullerene (200), solid lipid nanoparticles (SLNs) encompassing BBR by the spray-drying method (201), combining it with p-gp inhibitors (such as tariquidar and tetrandrine) (196, 198), and modification to berberine organic acid salts (BOAs) (168). However, the above-mentioned strategies were mostly tested in animals and must be demonstrated in human clinical trials.

In recent years, increasing evidence has suggested that nanoparticle (NP)-based delivery systems could be a great potential strategy to improve the therapeutic effects of BBR as an anticancer drug (202, 203). NPs, with a small diameter of 5–200 nm, are important artificial components of nanomedicine and play a key role as a therapeutic drug delivery system. NPs can effectively detect cancer and elucidate cancer-related mechanisms, especially as therapeutic delivery vehicles (204). NPs have unique physical and chemical properties, as well as powerful biological functions. They can effectively protect cargo from the degradation of enzymes and mechanisms (205). Several categories of nanoscale drug delivery vehicles have been demonstrated, and some of them have been applied in nanomedicine for tumor management, such as liposomes, carbon nanotubes, hydrogels, polymeric nanoparticles, and magnetic nanoparticles (206). BBR-loaded polymeric NPs (polyamidoamine dendrimers, chitosan NPs, and dextran NPs), metal NPs (iron-oxide NPs and mesoporous silica NPs), lipid NPs (liposomes), and carbon NPs (carbon dots and graphene NPs) have been revealed to have potential antitumor effects in different trials and experiments (203). Nonetheless, further studies regarding the efficacy of BBR in combination with different conventional anticancer drugs will pave the way for the use of BBR as a component of cancer chemotherapy.

## Conclusion and Future Challenges

Berberine exerts widespread pharmacological activities in different disorders. In this review, the functions of BBR are systematically explored and the antitumor effects on CRC, as well as the underlying mechanisms, are delineated. This review confirms that BBR has antibacterial, anti-inflammatory, antioxidant, antitumor, and hypoglycemic effects and cardiovascular and cerebrovascular protective functions. Furthermore, BBR exerts antitumor effects in CRC by modulating miRNA, lncRNA, and mRNA expression, inducing cell cycle arrest, inhibiting cell proliferation, stimulating cell apoptosis, and suppressing inflammation and oxidative stress *via* several signaling pathways. In addition to the above-mentioned functions, BBR has also revealed powerful antitumor activity in CRC by modulating the gut microbiota and mucosal barrier, which is good news for the search for natural antitumor drugs; however, to date, studies of the antitumor effects of BBR have been mainly performed in *in vitro* and a few *in vivo* models. Hence, *in vivo* studies, especially human studies, are warranted to further elucidate and confirm the therapeutic effects of BBR. Moreover, innovative approaches to adjust the BBR structure into more promising derivatives with stronger antitumor effects or to synergize with other chemotherapeutic drugs to improve the anticancer effects of BBR are needed. In summary, BBR is a safe, inexpensive, and effective long-term application for the treatment of cancers.

## Author Contributions

YS conceived the project and drafted the manuscript. XJ and ZJ performed research studies. MJ collected background information. All authors reviewed and approved the manuscript.

## Conflict of Interest

The authors declare that the research was conducted in the absence of any commercial or financial relationships that could be construed as a potential conflict of interest.

## Publisher's Note

All claims expressed in this article are solely those of the authors and do not necessarily represent those of their affiliated organizations, or those of the publisher, the editors and the reviewers. Any product that may be evaluated in this article, or claim that may be made by its manufacturer, is not guaranteed or endorsed by the publisher.
